# MD-DATA: the legacy of the ABC Consortium

**DOI:** 10.1007/s12551-024-01197-3

**Published:** 2024-05-07

**Authors:** Adam Hospital, Modesto Orozco

**Affiliations:** 1 Institute for Research in Biomedicine (IRB Barcelona), The Barcelona Institute of Science and Technology, Baldiri Reixac 10-12, 08028 Barcelona, Spain; 2 Department of Biochemistry and Biomedicine, University of Barcelona, 08028 Barcelona, Spain

## Abstract

The ABC Consortium has been generating nucleic-acids MD trajectories for more than 20 years. This brief comment highlights the importance of this data for the field, which triggered a number of critical studies, including force-field parameterization and development of new coarse-grained and mesoscopic models. With the world entering into a new data-driven era led by artificial intelligence, where data is becoming more essential than ever, the ABC initiative is leading the way for nucleic acid flexibility.

## Comment

Theoretical approaches to science aim to reduce the impact of serendipity on the advance of our knowledge. However, serendipity often guides the evolution of theoretical sciences. The Ascona B-DNA Consortium (ABC) is an excellent example of how new and unpredicted research objectives emerge when looking for very specific information. Thus, the first ABC round aimed to study the tetramer-dependent properties of B-DNA (Beveridge et al. 2004; Dixit et al. 2005), but unexpectedly the project helped to improve force-fields (FF; (Pérez et al. 2007)). The second round (Pasi et al. 2014) helped to describe bimodality in certain steps, but also resulted in the development of last-generation FFs (Ivani et al. 2016; Zgarbová et al. 2015). The third round aimed to reproduce DNA polymorphism (Dans et al. 2019), but as a side product, the analysis of data led to the description of the kinetics of DNA transitions and the development of a myriad of coarse-grained and mesoscopic models (Walther et al. 2020; López-Güell et al. 2023), which made it possible to move to the chromatin scale (Buitrago et al. 2021). We cannot predict what the impact of the ongoing HexABC project will be beyond characterizing the properties of the 2080 unique hexamers of DNA. The only clear statement that we can make is that stored ABC data will be crucial for it.

**Fig. 1:**
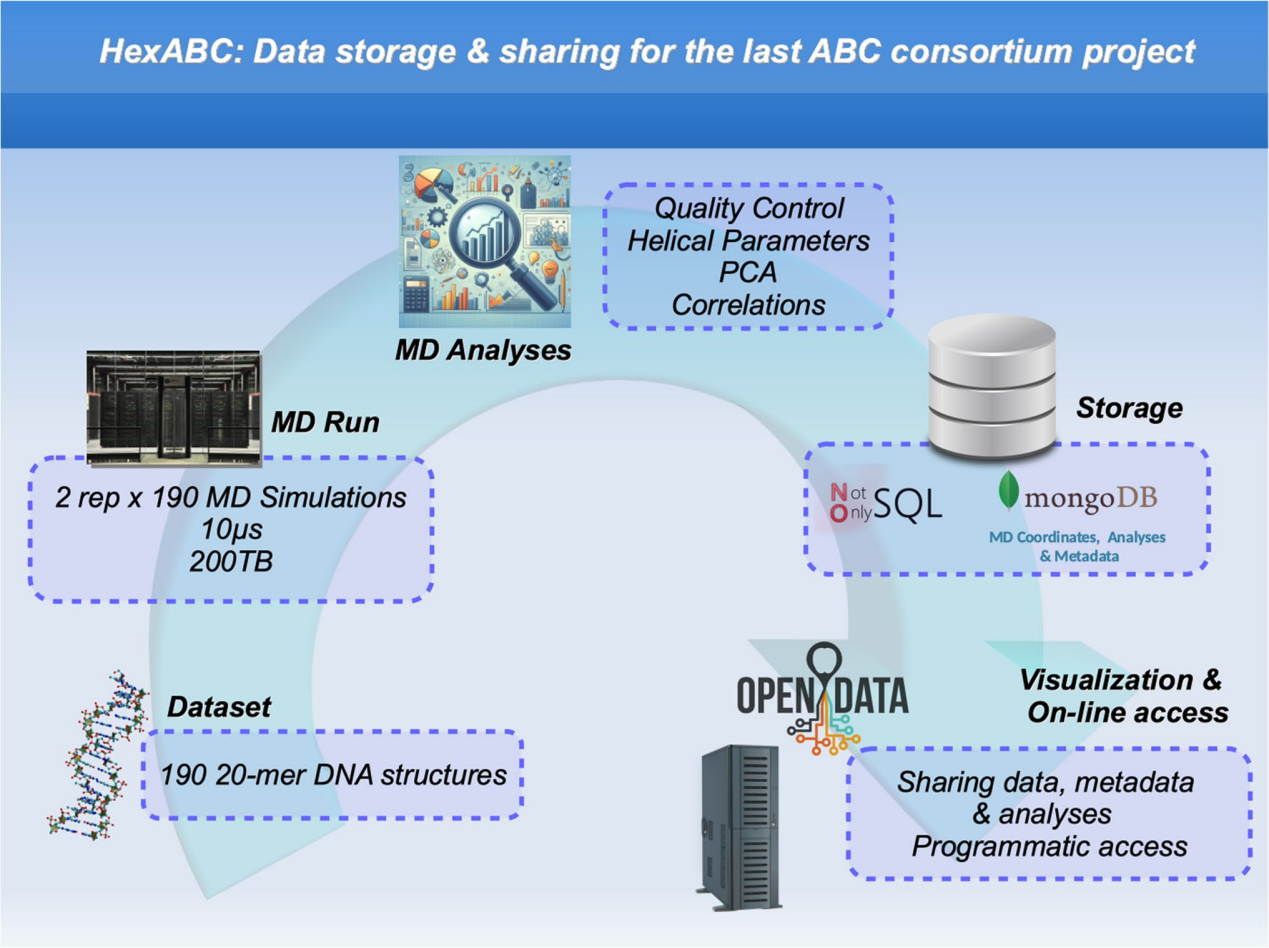
The main flow of HexABC data production and storage

Theoretical science is moving from an algorithm-based to a data-driven paradigm. Artificial intelligence methods are anxiously waiting for high-quality data to derive predictive models (Barissi et al. 2022). In this new scenario, we should be careful in reporting MD data with FAIR (findable, accessible, interoperable, and reusable) standards, providing provenance of the trajectories obtained using community-accepted simulation standards and stored after passing severe quality controls (Hospital et al. 2020). We should be prepared to solve computational challenges beyond mere CPU usage and closer to those faced by data-intensive sciences. ABC is pioneering the field: the HexABC consortium has generated 380 validated trajectories covering the 2080 unique DNA hexamers obtained using community-accepted standards. Performing such simulations has been a major effort for the 13 groups involved, but the greatest challenge has been to move around 200 TB of data from production sites to the datacenters in Utah and Barcelona, checking the integrity of the trajectories and detecting potential artefacts in the simulations that require human inspection. Analyzing 500,000 files (200 TB) and storing all the information in a NoSQL database with remote programmatic access represent an effort comparable to that of obtaining the trajectories. However, the final result: a validated database of B-DNA simulations will represent the best legacy of the ABC consortium (Fig. 1).

## Data Availability

Dataset generated by the ABC consortium and mentioned in the manuscript file will be made available as open data via the MDDB repository (https://mddbr.eu/).
